# Targeting MAP3K19 prevents human lung myofibroblast activation both *in vitro* and in a humanized SCID model of idiopathic pulmonary fibrosis

**DOI:** 10.1038/s41598-019-56393-z

**Published:** 2019-12-24

**Authors:** Isabelle C. Jones, Milena S. Espindola, Rohan Narayanan, Ana L. Coelho, David M. Habiel, Stefen A. Boehme, Tai Wei Ly, Kevin B. Bacon, Cory M. Hogaboam

**Affiliations:** 1 Department of Medicine, Cedars-Sinai Medical Center, Los Angeles, CA 90048 USA; 2 Axikin Pharmaceuticals, Inc., San Diego, CA USA

**Keywords:** Respiratory tract diseases, Drug development

## Abstract

Idiopathic Pulmonary Fibrosis (IPF) is a disease with a devastating prognosis characterized by unrelenting lung scarring. Aberrant activation of lung fibroblasts is a key feature of this disease, yet the key pathways responsible for this are poorly understood. Mitogen-activated protein kinase, kinase, kinase- 19 (MAP3K19) was recently shown to be upregulated in IPF and this MAPK has a key role in target gene transcription in the TGF-β pathway. Herein, we further investigate the role of MAP3K19 in cultured normal and IPF fibroblasts and in a humanized SCID mouse model of IPF employing both short interfering (si) RNA and novel small-molecule inhibitors directed at this kinase. Targeting MAP3K19 had significant inhibitory effects on the expression of both alpha smooth muscle actin and extracellular matrix in cultured human IPF fibroblasts. Quantitative protein and biochemical assays, as well as histological analysis, showed that MAP3K19 was required for the development of lung fibrosis in SCID mice humanized with IPF lung fibroblasts. MAP3K19 was required for IPF myofibroblast differentiation, and targeting its activity attenuated the profibrotic activity of these cells both *in vitro* and in an adoptive transfer SCID model of pulmonary fibrosis.

## Introduction

IPF is a devastating disease, characterized by the unrelenting spread of lung fibrosis in a honeycomb pattern throughout the lung parenchyma and a median survival of 3–5 years after diagnosis^1^. IPF patients exhibit symptoms such as a persistent cough and dyspnea, and lung function deterioration continues in these patients to the point of respiratory failure^1^. The pathogenesis of IPF is still unclear but in recent years consensus has focused toward a hypothesis that this disease is the result of repeated injury and/or genetic predisposition to dysfunction in alveolar type-2 epithelial cells (AEC)^2^. Sources of AEC injury include smoking, particulate inhalation, viral infections, and gastroesophageal reflux^2^. In response of AEC injury, fibroblasts are recruited to the site of injury and the recruited cells are subsequently activated via TGB-β to become myofibroblasts, which secrete excessive amounts of extracellular matrix (ECM) proteins^3^. Current approved therapeutics for IPF include nintedanib and pirfenidone (i.e. standard-of-care (SOC) drugs), and these therapeutics appear to have direct and indirect effects on myofibroblast activation^4^. However, adverse side effects and the failure of these drugs to halt progression in IPF highlights the need for a more effective treatment(s)^5,6^.

MAP3K19 (also referred also as RCK and YSK-4) is a conserved stress-induced protein, which is upregulated in alveolar and interstitial macrophages, bronchial epithelial cells, and type II pneumocytes in COPD and IPF^7,8^. Mechanistically, MAP3K19 appears to modulate TGF-beta signaling via the translocation (or shuttling) of activated Smad2 and Smad3 into the cell nucleus^8^. In a mouse model of smoke-induced emphysema, both an anti-MAP3K19 siRNA and a small-molecule inhibitor directed at this MAPK inhibited inflammation and improved airspace integrity^7^. Targeting MAP3K19 also strongly inhibited bleomycin-induced pulmonary fibrosis when administered either prophylactically or therapeutically^8^. In the current study, we report the effects of both siRNA and novel small-molecule inhibitors of MAP3K19 in primary human IPF fibroblasts and a SCID mouse model of IPF induced by the intravenous injection of IPF fibroblasts^9,10^.

## Methods

### Human lung samples

The current study was reviewed and approved by an Institutional Review Board at the University of Michigan Medical School (Ann Arbor, MI, USA). Informed consent was obtained from all patients who participated in the study described herein. Methods pertaining to patient samples were all conducted in accordance with all relevant guidelines and regulations. Patients were evaluated before undergoing fiberoptic bronchoscopy and clinical evaluations included chest radiography, lung function measurements, and thin section computed tomography^11^. Patient characteristics have been previously published in detail^12^.

### Human lung fibroblast isolation

Lung fibroblasts were derived from surgical lung biopsies (SLBs) from patients who exhibited slowly or rapidly progressing IPF (slow IPF and rapid IPF, respectively) over the first year after diagnosis^12^. Primary normal lung fibroblasts were derived from non-fibrotic lung samples lacking any evidence of disease. Histologically-proven IPF and normal lung samples were dissociated mechanically as previously described in detail^13^. Briefly, SLBs were mechanically dispersed in 150 cm^3^ flasks (Corning), combined with Dulbecco’s modified Eagle Medium (Lonza), containing 15% fetal bovine serum (Cell Generation), 0.1% Pen/Strep/Amphotericin B (10,000 U of penicillin/ml, 10,000 μg of streptomycin/ml, and 25 μg Amphotericin B/ml) (Lonza), 0.1% (29.20 mg/mL) L-glutamine (Corning), and Primocin at 100 μg/ml (Invivogen). Isolated fibroblasts were incubated at 37 °C in 10% CO_2_ until confluency was reached. The cells were passaged 3 to 4 times before use in the experiments described herein. In the present study, we analyzed a total of 4 normal fibroblast lines, 7 fibroblast lines derived from IPF patients who showed slow progression, and 7 fibroblast lines derived from IPF patients who showed rapid progression.

### Small molecule inhibitors of MAP3K19

#### AXP1741

Molecular mass is 302.34 g/mol. *In vitro* IC50 of this first-generation inhibitor directed against MAP3K19 was determined to be 177 nM. Testing this MAP3K19 inhibitor in a panel of 358 kinases, AXP1741 was found to partially inhibit PDGFRA and B, and YES1 at 10 μM. In PK studies with this compound the following was observed: Cmax 42 ng/ml; T1/2 32 hour; AUC 1163 μg*hr/L. Finally, AXP1741 was found to be efficacious in an A549 cancer xenograft model at 10 mg/kg in mouse (unpublished findings).

#### AXP2132

Molecular mass is 436.52 g/mol. The *in vitro* IC50 of this second-generation compound directed against MAP3K19 was determined to be low, at 435 nM, and the compound possessed sub-optimal PK properties so was not characterized further.

#### AXP2258

Molecular mass 408.46 g/mol. *In vitro* IC50 of this third-generation inhibitor directed against MAP3K19 was determined to be 8.7 nM (selectivity index 0.028, with inhibition of JAK1 and JAK2 being considered relevant off-target activity)). In PK studies with this compound the following was observed: Cmax 351 ng/ml; T1/2 2 hour; AUC 2050 μg*hr/L Finally, AXP2258 was also found to be highly efficacious in an A549 cancer xenograft model at 10 mg/kg and its effect was additive with Nexavar (unpublished findings).

### SCID mouse model of IPF

Female, C.B-17-scid-beige (C.B-17SCID/bg; 6 to 8 weeks old) were purchased from Taconic Biosciences (Germantown, NY), and maintained in barrier facility at the University of Michigan Medical School. All methods were performed in accordance with guidelines and regulations for animal care and use at the University of Michigan School of Medicine. All mice had access to autoclaved water and pelleted chow diet. All procedures with C.B-17SCID/bg mice were performed in a sterile environment and these procedures were approved by an animal care and use committee at the University of Michigan Medical School. This adoptive transfer model of lung fibrosis is initiated by the intravenous introduction of cultured IPF fibroblasts^9,10,12,14^. Cultures of an IPF fibroblast line with the highest transcript expression of MAP3K19 (line S117) were grown in 150 cm^2^ tissue culture flasks, and trypsinized to generated single cell suspensions. These isolated cells were then labeled with PKH26, as previously described^9^, and the labeled fibroblasts were transferred to a phosphate-buffered saline (PBS) solution at a concentration of 2 × 10^6^ cells/ml. Five hundred (500) μl of suspended fibroblasts was injected into the tail vein of each C.B-17SCID/bg mice. Groups of five C.B-17SCID/bg/group received one of the following at indicated times and doses following human fibroblast injection: (1) siRNA directed against human MAP3K19 (human siRNA3 5′-AGCATTGGTTGTACTGTGTTT-3′; mouse siRNA3 AGAGUGGUUGAGCGAGAUUTT (Qiagen)); both administered at 5 mg/kg intranasally every other day starting at either day 14 or 30 after human IPF fibroblast injection; (2) non-sense siRNA (human 5′-TCCATAACGCGTATACTCGAC-3′; mouse ACUAAGUACGUCGUAUUACTT (Qiagen)) both administered at 5 mg/kg intranasally every other day starting at either day 14 or 30 after human IPF fibroblast injection; (3) PBS (20 μl intranasally) every other day starting at either day 14 or 30 after human IPF fibroblast injection; (4) interleukin-13/Pseudomonas exotoxin chimeric protein (IL13-PE; we have observed the anti-fibrotic effects of this chimeric protein in other pulmonary fibrosis models^15,16^; 1 μg/dose in 20 μl intranasally) every other day starting at either day 14 or 30 after human IPF fibroblast injection; (5) vehicle used to suspend compounds (40% solutol + 10% ethanol + 50% H_2_O) orally; (6) AXP1741 (10 mg/kg) daily orally; (7) AXP2132 (10 mg/kg) daily orally; or (8) a multi-JAK inhibitor Tofacitinib (1 mg/kg) daily orally (as control for the off-target activity of AXP2258 directed at JAK1 and JAK3); and (9) AXP2258 (1 mg/kg) daily orally. All mice were euthanized by anesthesia overdose at days 60 or 63 after injection of human IPF fibroblasts, and whole lung tissue was dissected and used for subsequent analysis of histologic, molecular, and protein parameters.

### Histological analysis

Following euthanasia, right lobes of each C.B-17SCID/bg mouse were dissected and infused with 10% formalin solution, then placed into a fresh formalin solution for 24 hours. Lungs were paraffin-embedded using a standard technique. Lungs were sectioned at 5 μm and stained for hematoxylin and eosin, and Masson’s trichrome for architecture analysis.

### Transcript expression analysis

RNA was isolated from cultured normal and IPF (both from slow and rapid progressing patients) fibroblasts as well as whole-lung samples using RNeasy kits (Qiagen). Isolated RNA was reverse transcribed into cDNA using SYBR Green PCR Master Mix (Applied Biosystems) and 5 ng of cDNA was used for transcript quantification via RT-PCR using a ViiA 7 (Applied Biosystems). Oligonucleotides primers were designed and validated by Integral DNA Technologies. MAP3K19 primers were obtained from Axikin: sense primer: 5′AATGGCACCCACAGTGACATGCTT3′; antisense primer: 5′CCCTCGGTGTGCTCCGATCTAAAA3′. The amplification efficiencies were determined for target genes and normalized to GAPDH. Delta Ct was used for comparative analysis. Commercially available primer and probe sets for both human collagen 3 and fibronectin was obtained from Applied Biosciences (Thermo Fisher). Fold change and gene expression were analyzed with Data Assist software (Life Technologies).

### ELISA

Alpha smooth muscle actin (αSMA) and β-tubulin proteins were measured using an in-cell ELISA technique. Cell-free supernatants (50 μl) from cultured fibroblasts were used to measure collagen type 1 levels, using a direct ELISA, as previously described^17^. β-tubulin values were used to normalize values of αSMA and Collagen 1.

### Hydroxyproline assay

Total lung collagen concentration was calculated using a technique previously described^10^. Briefly, whole-lung tissue was homogenized (Pro Scientific, Micro Homogenizer). Five hundred (500) μl of homogenate samples was hydrolyzed in 1 ml of 6 N HCl, for 8 h at 120 °C. For each sample, 5 μl of sample combined with 5 μl of citrate/acetate buffer (5% citric acid, 7.2% sodium acetate, 3.4% sodium hydroxide, and 1.2% glacial acetic acid, pH 6) and 100 μl of chloramine-T solution (282 mg chloramine-T, 2 ml of n-propanol, 2 ml of d-H_2_O, and 16 ml of citric acid buffer). Solution samples then were incubated at room temperature for 20 min, after which 100 μl of Ehrlich’s solution (Aldrich) was added. After incubating in Ehrlich’s solution for 15 min at 65 °C, the samples were analyzed with a Beckman DU 640 spectrophotometer at a wavelength of 550 nm. Sample hydroxyproline concentration was calculated from a standard curve (0–100 μg/ml).

### Statistical analyses

All statistical analyses were conducted using GraphPad Prism 7. Data are presented as mean ± SEM. Statistical significance, when comparing two groups, was determined using nonparametric t-tests (either Mann-Whitney or Kolmogorov-Smirnoff) to compare differences between IPF (Stable/Rapid) groups and normal lung groups. When comparing more than two groups, Ordinary One-way ANOVA tests were used. P values considered significant; *p < 0.05, **p < 0.01, ***p < 0.001.

## Results

### IPF biopsies and fibroblasts have increased expression of MAP3K19 transcript compared with normal biopsies and fibroblasts

**Figure 1:**
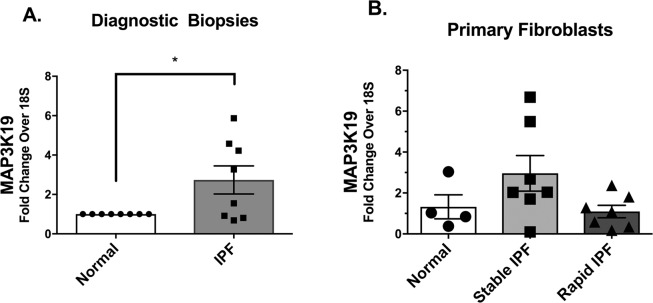
Increased Transcript Levels of MAP3K19 in IPF Biopsies and Fibroblasts. Fold changes in MAP3K19 transcript levels comparing normal lung samples (n = 8) to that of IPF diagnostic lung biopsy samples (n = 8). (**A**) Fold changes in MAP3K19 transcript expression comparing cultured normal lung fibroblasts (n = 4 lines) with fibroblasts derived from stable or slow progressing IPF patients (n = 7 lines) and fibroblasts derived from rapid progressing IPF patients (n = 7 lines). (**B**) Data are mean ± SEM. *P ≤ 0.05 compared with normal biopsies.

MAP3K19 transcript levels in diagnostic biopsies from IPF patients were significantly elevated when compared to normal lung samples, (Fig. 1A). IPF fibroblasts from patients who exhibited slow progression or stable disease (i.e. stable IPF fibroblasts) expressed the highest transcript expression of MAP3K19 compared with normal fibroblasts or IPF fibroblasts derived from IPF patients who exhibited rapid progression of disease (rapid IPF fibroblasts) (Fig. 1B). Together, these data suggested that MAP3K19 was increased in IPF.

### Hydroxyproline, fibronectin, and collagen III were reduced by MAP3K19-targeting siRNA in a humanized C.B-17SCID/bg model of IPF

**Figure 2:**
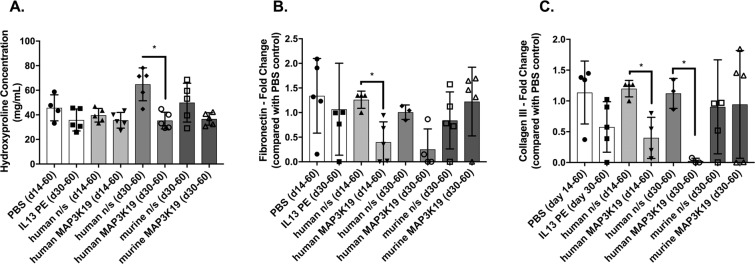
Short interfering RNA directed against human MAP3K19 reduced fibrosis in a humanized SCID model of IPF. Line S117 IPF fibroblasts were injected intravenously into C.B-17SCID/bg mice as previously described in detail^8^. At day 14 after human IPF fibroblast injection, groups of 5 humanized C.B-17SCID/bg mice received either PBS control, human scrambled or nonsense (n/s) siRNA, or human MAP3K19 siRNA. At day 30 after human IPF fibroblast injection, other groups of 5 humanized C.B-17SCID/bg mice received IL13-PE, human n/s siRNA, human MAP3K19 siRNA, murine n/s siRNA, or murine MAP3K19 siRNA. At day 60 after human fibroblast injection, all groups were euthanized, and lung samples were removed and processed for hydroxyproline (n = 5). (**A**) Fibronectin transcript (n = 5) (**B**) and collagen 3 transcript expression (**C**) (n = 5), were measured through RT-qPCR analysis. Data are mean ± SEM. *P ≤ 0.05 compared with appropriate control group as indicated.

To address the role of MAP3K19 role in fibrosis *in vivo*, IPF fibroblasts were injected into C.B-17SCID/bg mice as previously described in detail^9,12^. Beginning at either days 14 (during the development of fibrosis^9^) or 30 (when fibrosis is established^9^) after IPF fibroblast injection (line S117A), C.B-17SCID/bg mice received PBS, IL13-PE, scrambled siRNA (n/s), or siRNA targeting either mouse or human MAP3K19 by intranasal delivery every other day until day 60 after human fibroblast injection. As shown in Fig. 2, humanized C.B-17SCID/bg that received the siRNA directed against human (but not mouse) MAP3K19 from days 14–60 had no significant changes in hydroxyproline content (Fig. 2A) but showed a significant reduction in fibronectin and collagen 3 transcripts compared to humanized C.B-17SCID/bg that received n/s RNA control RNA (Fig. 2B,C). Further, mice receiving human-specific MAP3K19 siRNA from days 30–60 after IPF fibroblast injection had significantly less hydroxyproline (Fig. 2A), and a reduction in fibronectin and collagen 3 transcripts (Fig. 2B,C) compared with humanized C.B-17SCID/bg that received n/s RNA control RNA. Thus, the therapeutic targeting of human MAP3K19 via a siRNA approach significantly reduced pulmonary fibrosis as assessed both using biochemical and transcript analysis approaches in a humanized C.B-17SCID/bg model of IPF.

### Small molecule inhibitors of MAP3K19 reduced hydroxyproline and collagen 3 in a humanized C.B-17SCID/bg model of IPF

**Figure 3:**
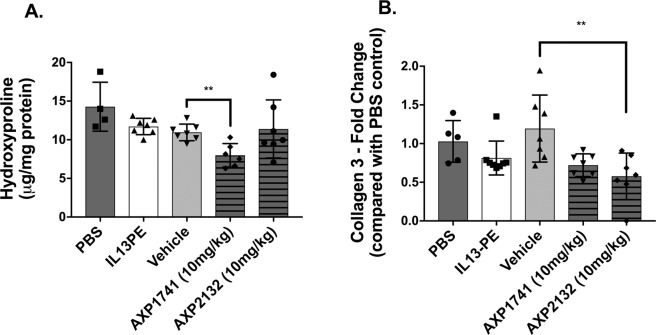
AXP1741 and AXP2132 significantly reduced markers of pulmonary fibrosis in a humanized SCID model of IPF. At day 0, groups of SCID mice (n = 5) were intravenously injected with 1 × 10^6^ fibroblasts and treated as described in the Methods and Materials. At day 35, humanized SCID mice were treated with one of PBS, IL13-PE, drug vehicle, AXP1741, or AXP2132 (both at 10 mg/kg). On day 60 after human fibroblast injection, all groups of humanized SCID mice were euthanized and lung samples were processed for hydroxyproline (**A**), or RNA for determination of collagen 3 transcript levels using RT-qPCR methods (**B**). Data are mean ± SEM. **P ≤ 0.01 compared with the appropriate control group of humanized SCID mice as indicated.

In the next series of experiments, two small-molecule inhibitors of MAP3K19, namely AXP1741 and AXP2132, were tested in a humanized mouse model of IPF. In these studies, stable IPF fibroblasts (line S117A) were injected into C.B-17SCID/bg mice and beginning at day 35 after fibroblast injection, groups of 5 mice received one of the following every other day until day 63 after fibroblast injection: PBS (intranasally), IL13-PE (500 ng/dose intranasally), compound vehicle (100 μl orally), AXP1741 (10 mg/kg orally) and AXP2132 (10 mg/kg orally). Although AXP1741 therapy significantly reduced hydroxyproline compared with the oral vehicle control (Fig. 3A), it was AXP2132 that had the significant/greatest effect on transcript levels of collagen 3 compared with the oral vehicle control (Fig. 3B). Analysis of Masson’s trichrome-stained whole lung sections from C.B-17SCID/bg injected intravenously with primary IPF fibroblasts revealed that therapeutic treatment with AXP1741 (Suppl. 1B) but to a lesser extent AXP2132 (Suppl. 1C) restored both the lung architecture and reduced collagen staining compared to the vehicle control (Suppl. 1A). Together, these data demonstrated that targeting MAP3K19 using a small-molecule approach therapeutically reduced pulmonary fibrosis in a humanized C.B-17SCID/bg mouse model of IPF.

### *In vitro* targeting of MAP3K19 with AXP2258 enhanced the inhibitory effects of pirfenidone in primary normal, slow, and rapid IPF fibroblasts

**Figure 4:**
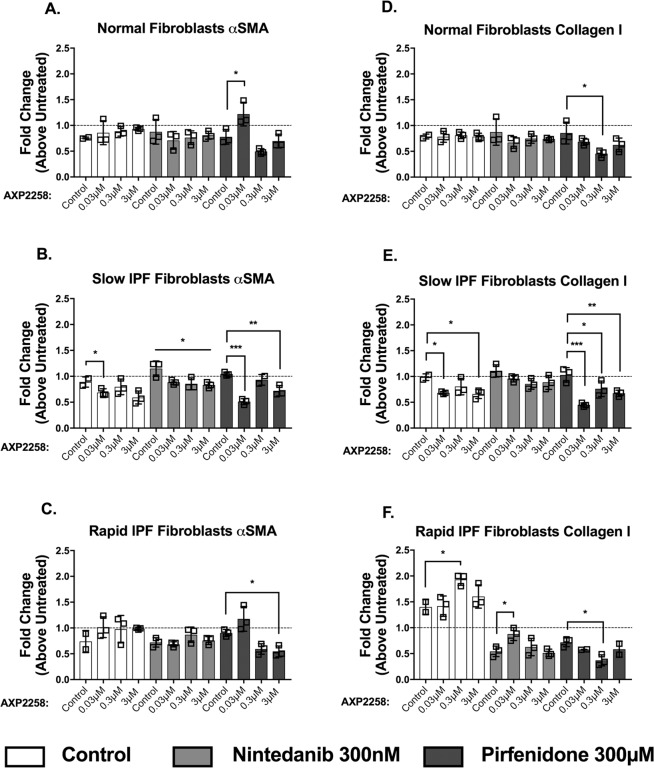
Effects of the MAP3K19 inhibitor AXP2258 alone and in combination with either pirfenidone or nintedanib on α smooth-muscle actin and collagen I expression in primary human lung fibroblasts. Fibroblasts of normal lung (**A,D**) (n = 3), slow IPF (**B,E**) (n = 3), and rapid IPF (**C,F**) (n = 3) patients were plated in 96-well tissue culture plates at a cell density of 5 × 10^4^/well in sets of triplicate wells for each treatment (n = 3), and plates were incubated for 24 hours before treatments were added. Treatments for fibroblasts were as follows: untreated (i.e. media alone), DMSO vehicle control for all compounds, AXP2258 (at 0.03 μM, 0.3 μM, or 3 μM) ± BIBF-1200 (300 nM), ±pirfenidone (300 μM). Twenty-four hours later, αSMA and collagen 1 were measured by ELISA and normalized to β-tubulin values. AXP2258 alone (open bars) were compared to DMSO control. AXP2258 (shaded bars) with BIBF-1200 were compared to the BIBF-1120 (nintedanib) control. AXP2258 (black bars) with pirfenidone were compared to the pirfenidone treatment control. All treatments are represented as fold-change compared to untreated, which is represented by the dotted line. Data are mean ± SEM. *P ≤ 0.05; **P ≤ 0.01; ***P ≤ 0.001 compared with the appropriate control treatment as indicated.

We undertook additional studies with a more potent inhibitor called AXP2258, and also explored the combinatorial effects of AXP2258 and either BIBF1120 (i.e. nintedanib) or pirfenidone on human lung fibroblast activation via the measure of both αSMA and collagen 1 protein. AXP2258 had no effect on generation of either αSMA and collagen 1 protein in normal lung fibroblasts compared with the DMSO vehicle control (Fig. 4A,D). However, compared with the DMSO vehicle control groups, we observed that AXP2258 had statistically significant inhibitory effects on both αSMA and collagen 1 protein levels in cultured IPF fibroblasts from a slow IPF progressor (Fig. 4B,E). AXP2258 alone (at 0.3 μM but not at other concentrations) significantly increased αSMA protein levels in rapid IPF lung fibroblasts compared with the DMSO control (Fig. 4C,F).

The combination of AXP2258 and the SOC compound nintedanib had no effect on either myofibroblast protein marker compared with the nintedanib alone group in cultured normal lung fibroblasts (Fig. 4A,D). When AXP2258 was added with SOC compound pirfenidone in cultured normal lung fibroblasts, significantly increased αSMA protein expression (AXP2258 at 0.03 μM) and significantly decreased collagen 1 protein expression (AXP2258 at 0.3 μM) were observed when compared with the pirfenidone alone control group (Fig. 4A,D). When AXP2258 was added with nintedanib in cultured slow IPF lung fibroblasts, statistically significant reductions in αSMA but not collagen 1 protein levels were observed compared with the nintedanib alone control group (Fig. 4B,E). When AXP2258 was added with pirfenidone in cultured slow IPF lung fibroblasts, significantly decreased αSMA and collagen 1 protein were observed compared with the pirfenidone alone control group (Fig. 4B,E). When AXP2258 was added with nintedanib in cultured rapid IPF lung fibroblasts, there was no effect on αSMA protein and significantly increased collagen 1 protein generation (AXP2258 at 0.03 μM) compared with the nintedanib alone group (Fig. 4C,F). The addition of AXP2258 and pirfenidone to cultures of rapid IPF fibroblasts resulted in significantly decreased levels of both αSMA protein and collagen 1 protein compared with the pirfenidone alone group (Fig. 4C,F). Thus, either targeting MAP3K19 alone (with AXP2258) or in combination with pirfenidone significantly modulated myofibroblast differentiation as determined by αSMA protein and collagen 1 protein levels in cell lines derived from patients who exhibited slow progression of IPF.

### AXP2258 significantly reduced biochemical and histologic parameters of fibrosis in a humanized C.B-17SCID/bg SCID model of fibrosis

**Figure 5:**
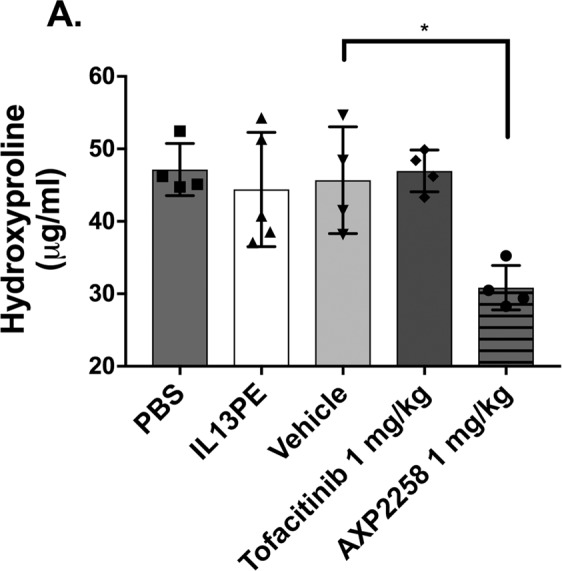
MAP3K19 inhibitor AXP2258 significantly reduced pulmonary fibrosis in a humanized SCID model of IPF. Line S117 (slow) IPF fibroblasts (n = 5) were injected intravenously into C.B-17SCID/bg mice as previously described in detail^8^. At day 35 after human IPF fibroblast injection, groups of 5 humanized C.B-17SCID/bg mice received either PBS control, IL13-PE, vehicle for compounds, a JAK inhibitor (Tofacitinib; 1 mg/kg), or AXP2258 (1 mg/kg). At day 63 after human fibroblast injection, all groups were euthanized, and lung samples were removed and processed for hydroxyproline. (**A**) Data are mean ± SEM. *P ≤ 0.05 compared with the appropriate control treatment as indicated.

**Figure 6:**
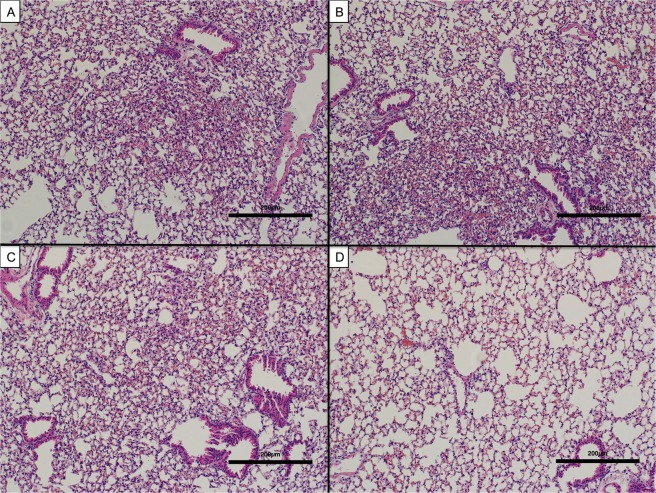
MAP3K19 inhibitor AXP2258 reduced lung fibrosis in a humanized SCID model of IPF. Line S117 (slow) IPF fibroblasts were injected intravenously into C.B-17SCID/bg mice as previously described in detail^8^. At day 35 after human IPF fibroblast injection, groups of 5 humanized C.B-17SCID/bg mice received either PBS control, IL13-PE, vehicle for compounds, a JAK inhibitor (Tofacitinib; 1 mg/kg), or AXP2258 (1 mg/kg). At day 63 after human fibroblast injection, all groups were euthanized, and lung samples were removed and processed using routine histologic techniques, and hematoxylin & eosin-stained slides were analyzed. Representative images are shown for the PBS-treated group (**A**), vehicle-treated group (**B**), Tofacitinib-treated group (**C**), and AXP2258-treated group. (**D**) Original magnification was 100X. Scale bars are shown at 200 µm.

**Figure 7:**
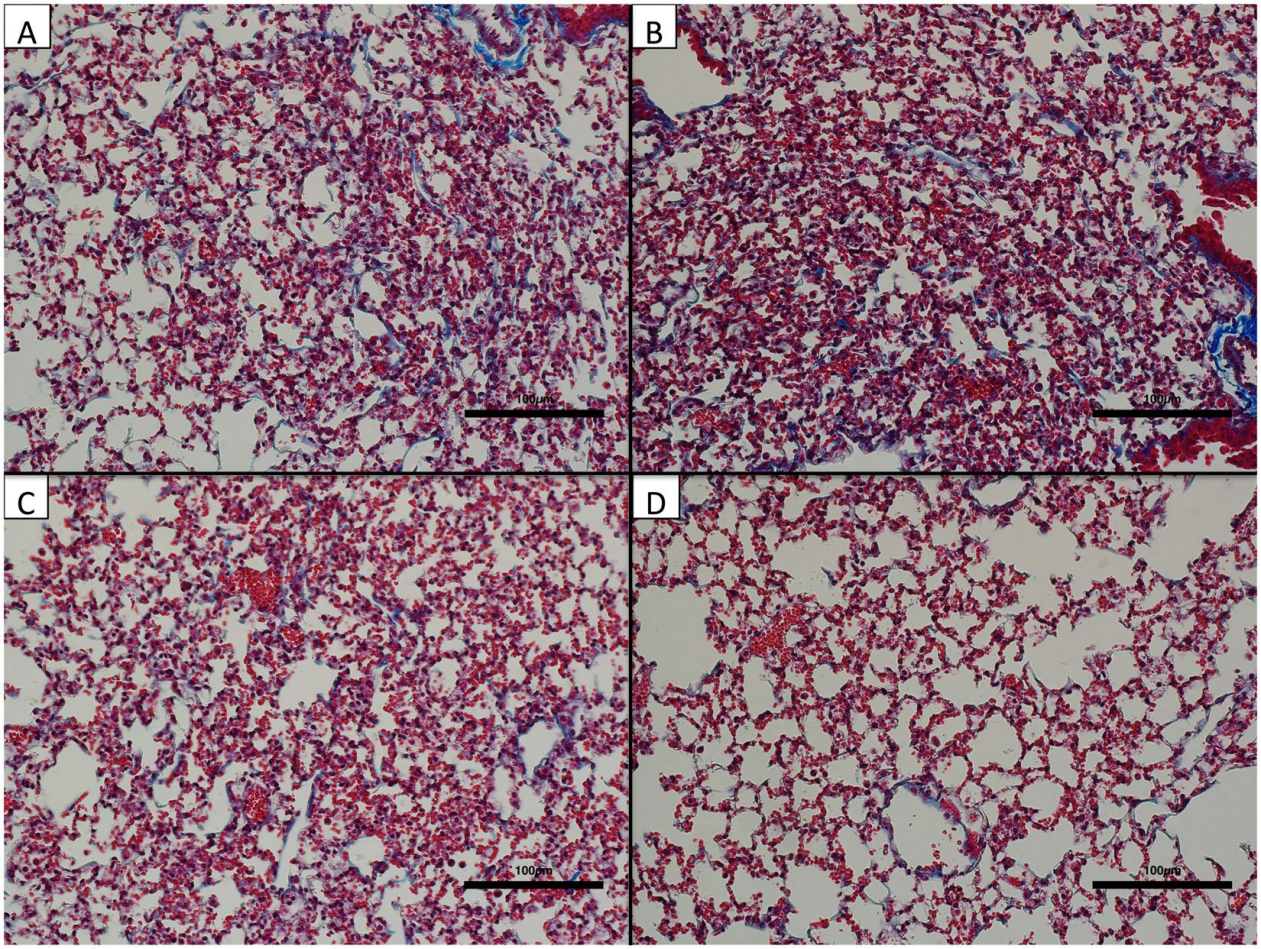
MAP3K19 inhibitor AXP2258 reduced trichrome Masson staining in a humanized SCID model of IPF. Line S117 IPF fibroblasts were injected intravenously into C.B-17SCID/bg mice as previously described in detail^8^. At day 35 after human IPF fibroblast injection, groups of 5 humanized C.B-17SCID/bg mice received either PBS control, IL13-PE, vehicle for compounds, a JAK inhibitor (Tofacitinib; 1 mg/kg), or AXP2258 (1 mg/kg). At day 63 after human fibroblast injection, all groups were euthanized, and lung samples were removed and processed using routine histologic techniques, and Masson’s trichrome-stained slides were analyzed. Representative images are shown for the PBS-treated group (**A**), vehicle-treated group (**B**), Tofacitinib-treated group (**C**), and AXP2258-treated group. (**D**) Original magnification was 100X. Scale bars are shown at 100 µm.

The last set of experiments addressed the therapeutic effects of a third inhibitor of MAP3K19, namely AXP2258, which was administered from days 35 to 63 in a C.B-17SCID/bg model of IPF. Compared with the appropriate controls and two other treatments (IL13-PE (1 μg/dose) and Tofacitinib (a JAK inhibitor; at 1 mg/kg)), AXP2258 (at 1 mg/kg) significantly reduced the total lung concentration of hydroxyproline at day 63 after fibroblast injection (Fig. 5). Histologic assessment of H/E-stained whole lung sections from C.B-17SCID/bg injected with stable IPF fibroblasts confirmed that there was markedly less lung consolidation in the AXP2258 therapy group (Fig. 6D) compared with PBS (Fig. 6A), compound vehicle (Fig. 6B), and Tofacitinib (Fig. 6C) groups. Histologic assessment of Masson’s trichrome-stained whole lung sections from C.B-17SCID/bg injected with stable IPF fibroblasts confirmed that there was markedly less collagen deposition in the AXP2258 therapy group (Fig. 7D) compared with PBS (Fig. 7A), compound vehicle (Fig. 7B), and Tofacitinib (Fig. 7C) groups. Together, these data demonstrate that AXP2258 was a potent inhibitor of the fibrotic response in a translational model of lung fibrosis.

## Discussion

It is hypothesized that repetitive and unrelenting injury to the lung is a major mechanism driving the fibrotic process in IPF^2^. Nintedanib and pirfenidone are two FDA-approved drugs that slow but do not stop progression in this disease^5,18,19^. This benefit is not without serious tolerance issues as evidenced by the debilitating side-effects IPF patients experience while taking either or both of these therapeutics^20^, thus providing the impetus to pursue other therapeutic strategies in IPF. Previous studies had indicated that targeting MAP3K19 in the context of experimental smoke-induced^7^ and bleomycin-induced^8^ lung injury and remodeling was therapeutic in both models via mechanisms involving inflammation and TGF-β-induced remodeling. Consequently, the aim of our study was to further explore the role of MAP3K19 in IPF and determine its role in primary human lung fibroblasts and in a C.B-17SCID/bg model of IPF. We show that MAP3K19 was highly expressed in IPF fibroblasts particularly those derived from IPF patients who exhibited stable or a slowly progressive IPF in the first year after diagnosis (as previously defined^12^). Targeting MAP3K19 both *in vitro* and *in vivo* markedly altered IPF fibroblast to myofibroblast differentiation and the maintenance of pulmonary fibrosis in C.B-17SCID/bg humanized with IPF fibroblasts. Thus, these data demonstrate that MAP3K19 modulates fibrosis via direct effects on primary human IPF fibroblasts.

Overall analysis of diagnostic biopsies from IPF patients indicated that there was an approximate 3-fold difference in MAP3K19 transcript expression between normal and IPF lung samples. Further exploration of the samples and cells from these samples showed that this increase was restricted to IPF patients who exhibited slow progression or more stable disease in the first year after diagnosis. The criteria we used to define slow and rapid decline in IPF are found in our previous study by Trujillo *et al*.^12^ and it incorporates both changes in both forced vital capacity (FVC) and diffusing co-efficient of carbon monoxide (D_L_CO) as discriminators in these IPF patient subgroups. Our *in vitro* analysis of slow IPF fibroblasts confirmed that MAP3K19 was upregulated in these cells. At present, it is not clear as to why the expression of MAP3K19 was higher and regulated in slow IPF fibroblasts and not in rapid IPF fibroblasts. Our previous studies have documented considerable differences between lung fibroblasts cultured from these subsets of IPF patients including differences in responses to hypomethylated DNA^12,21^, double-stranded RNA^22^, and interleukin-4 & 13 (IL-4/IL-13)^14^. For example, we have observed that slow IPF fibroblasts are less responsive to IL-4/IL-13 compared with rapid IPF fibroblasts^14,23^ and these findings were confirmed in the present study with the observation that the immunotoxin IL13-PE had no anti-fibrotic effects in the humanized SCID model of IPF induced by the introduction of stable IPF fibroblasts. In previous studies, we have observed that IL13-PE was selective and only targeted IPF cells with upregulated IL-13Rα2^16,24^, which is typically observed in rapid IPF fibroblasts. What dictates responsiveness and non-responsiveness to external signals as well as the differential nature of signaling pathways in rapid versus slow IPF remains an active area of investigation. Clearly, further work is required to better define the fibroblast-centric mechanisms that distinguish rapid from slow progression in IPF.

Our data suggest that MAP3K19 affects the synthetic properties of IPF fibroblasts to a greater extent compared with primary normal lung fibroblasts, particularly with respect to myofibroblast differentiation, which we defined in cultured fibroblasts as changes in both α-SMA and collagen 1 protein expression^25^. Specifically, when added alone to cultures of primary lung fibroblasts, AXP2258 (which was the most potent MAP3K19 small-molecule inhibitor tested herein) significantly inhibited the proteins levels of both myofibroblast markers in cultures of slow IPF fibroblasts compared with the DMSO control group. AXP2258 treatment alone either had no effect or increased α-SMA in cultures of normal and rapid IPF fibroblasts, respectively. Interestingly, the addition of pirfenidone with AXP2258 to cultures of slow and rapid IPF fibroblasts significantly inhibited α-SMA protein expression in these cells compared with pirfenidone treatment alone. Also, the addition of pirfenidone with AXP2258 to cultures of all three human fibroblast types examined in this study significantly inhibited collagen-1 protein expression in these cells compared with pirfenidone treatment alone in our *in vitro* assays. Unlike with AXP2258 combination with pirfenidone, the combination of AXP2258 with nintedanib only significantly decreased α-SMA protein expression compared with nintedanib treatment alone in cultures of slow IPF fibroblasts. Why there were differences between the effects of combined AXP2258 and the SOCs is not clear at the moment but differences in responses to drug + SOC combinations in IPF trials are beginning to appear in the literature^26^. Thus, AXP2258 treatment alone inhibited fibroblast to myofibroblast differentiation in fibroblasts from slow IPF patients but when this MAP3K19 inhibitor was combined with pirfenidone inhibitory effects on myofibroblast differentiation were observed in both slow and rapid IPF fibroblasts.

In a previous study employing a bleomycin-induced fibrosis model, it was shown that therapeutically dosing with AXP1741 at 10 mg/kg significantly reduced the fibrotic response to this injury^8^. In the present study, AXP1741 and another second-generation small-molecule inhibitor of MAP3K19, namely AXP2132, therapeutically modulated fibrosis in a humanized C.B-17SCID/bg mouse model initiated by the intravenous injection of stable IPF fibroblasts. More importantly, we noted that the clinical candidate AXP2258 exhibited the strongest inhibitory effect on the fibrosis parameters measured in this model. Although this compound has been shown to also target JAKs, treatment with the JAK inhibitor Tofacitinib had no effect on the pulmonary fibrosis observed in this humanized SCID model of IPF. The *in vivo* studies in humanized C.B-17SCID/bg mouse model were conducted using a stable IPF fibroblast line because fibroblast lines from this subtype of IPF showed the most consistent expression of MAP3K19. Interestingly, the anti-fibrotic effects of these two small molecule inhibitors in this model were similar to the *in vivo* effects of siRNA directed at human but not mouse MAP3K19. Although the mechanism via which MAP3K19 modulates fibrosis in the humanized SCID model is not presently clear, we speculate that MAP3K19 works via a TGF-β-dependent pathway, as previously described in the bleomycin model of murine lung fibrosis^8^. Thus, the exact mechanism via which MAP3K19 regulates fibroblast to myofibroblast transition in IPF patients requires further investigation particularly whether the targeting of MAP3K19 alters lung responsiveness to profibrotic mediators including several growth factors.

In conclusion, our studies demonstrate that MAP3K19 has a pro-fibrotic role in IPF. A review of studies addressing the targeting of the TGF-β/SMAD2/3 pathway in fibrosis highlights a consensus that the biggest problem with this approach relates to cell-specificity, *in vivo* reproducibility, and most importantly adverse side-effects^27^. MAP3K19 is unique in that it is highly specific to the lung and it is uniquely upregulated in chronic lung diseases with abnormal remodeling^8^.

## Supplementary information

Supplementary Figure 1
