# Supplementary material for: Targeting MAP3K19 prevents human lung myofibroblast activation both in vitro and in a humanized SCID model of idiopathic pulmonary fibrosis
Source: Sci Rep. 2019 Dec 24;9:19796. doi: 10.1038/s41598-019-56393-z (PMC6930295; doi:10.1038/s41598-019-56393-z)
Supplement: Supplementary file 1 — Supplementary Figure 1 [file 41598_2019_56393_MOESM1_ESM.pdf]

Supplementary Figure 1

# Targeting MAP3K19 prevents human lung myofibroblast activation both *in vitro* and in a humanized SCID model of idiopathic pulmonary fibrosis.

Isabelle C. Jones[1], Milena S. Espindola[1], Rohan Narayanan[1],
Ana L. Coelho[1], David M. Habiel[1], Stefen A. Boehme[2],
Tai Wei Ly[2], Kevin B. Bacon[2], Cory M. Hogaboam[1,*].
[1]Department of Medicine, Cedars-Sinai Medical Center, Los Angeles, CA, 90048
[2]Axikin Pharmaceuticals, Inc., San Diego, CA,

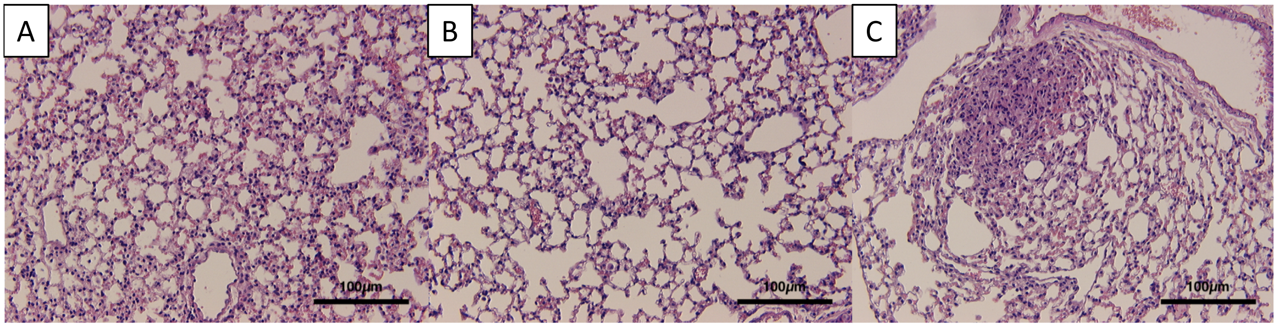


**Supplementary Figure 1**: Masson's trichrome-stained whole lung sections from C.B-17SCID/bg injected intravenously with human IPF fibroblasts. Humanized SCID mice were treated with compound vehicle (**A**), AXP1741 (10 mg/kg) (**B**), or AXP2132 (10 mg/kg)(**C**) in a therapeutic manner from days 35 to 63 after the human fibroblast injection.
